# Therapeutic potential of conduction system pacing as a method for improving cardiac output during ventricular tachycardia

**DOI:** 10.1007/s10840-024-01809-8

**Published:** 2024-04-22

**Authors:** Daniel Keene, Alejandra A. Miyazawa, Ahran D. Arnold, Akriti Naraen, Nandita Kaza, Jagdeep S. Mohal, David C. Lefroy, Phang Boon Lim, Fu Siong Ng, Michael Koa-Wing, Norman A. Qureshi, Nick W. F. Linton, Ian Wright, Nicholas S. Peters, Prapa Kanagaratnam, Matthew J. Shun-Shin, Darrel P. Francis, Zachary I. Whinnett

**Affiliations:** 1 Imperial College Healthcare NHS Trust, London, UK; 2 Imperial College London, National Heart and Lung Institute, London, UK; 3 National Heart and Lung Institute, Hammersmith Hospital, London, W12 0HS UK

**Keywords:** Ventricular tachycardia, VT, Hemodynamic, Mechanism, His bundle pacing, Conduction system pacing

## Abstract

**Background:**

Ventricular tachycardia (VT) reduces cardiac output through high heart rates, loss of atrioventricular synchrony, and loss of ventricular synchrony. We studied the contribution of each mechanism and explored the potential therapeutic utility of His bundle pacing to improve cardiac output during VT.

**Methods:**

Study 1 aimed to improve the understanding of mechanisms of harm during VT (using pacing simulated VT). In 23 patients with left ventricular impairment, we recorded continuous ECG and beat-by-beat blood pressure measurements. We assessed the hemodynamic impact of heart rate and restoration of atrial and biventricular synchrony. Study 2 investigated novel pacing interventions during clinical VT by evaluating the hemodynamic effects of His bundle pacing at 5 bpm above the VT rate in 10 patients.

**Results:**

In Study 1, at progressively higher rates of simulated VT, systolic blood pressure declined:

at rates of 125, 160, and 190 bpm, -22.2%, -42.0%, and -58.7%, respectively (ANOVA *p* < 0.0001). Restoring atrial synchrony alone had only a modest beneficial effect on systolic blood pressure (+ 3.6% at 160 bpm, *p* = 0.2117), restoring biventricular synchrony alone had a greater effect (+ 9.1% at 160 bpm, *p* = 0.242), and simultaneously restoring *both* significantly increased systolic blood pressure (+ 31.6% at 160 bpm, *p* = 0.0003). In Study 2, the mean rate of clinical VT was 143 ± 21 bpm. His bundle pacing increased systolic blood pressure by + 14.2% (*p* = 0.0023). In 6 of 10 patients, VT terminated with His bundle pacing.

**Conclusions:**

Restoring atrial and biventricular synchrony improved hemodynamic function in simulated and clinical VT. Conduction system pacing could improve VT tolerability and treatment.

**Graphical Abstract:**

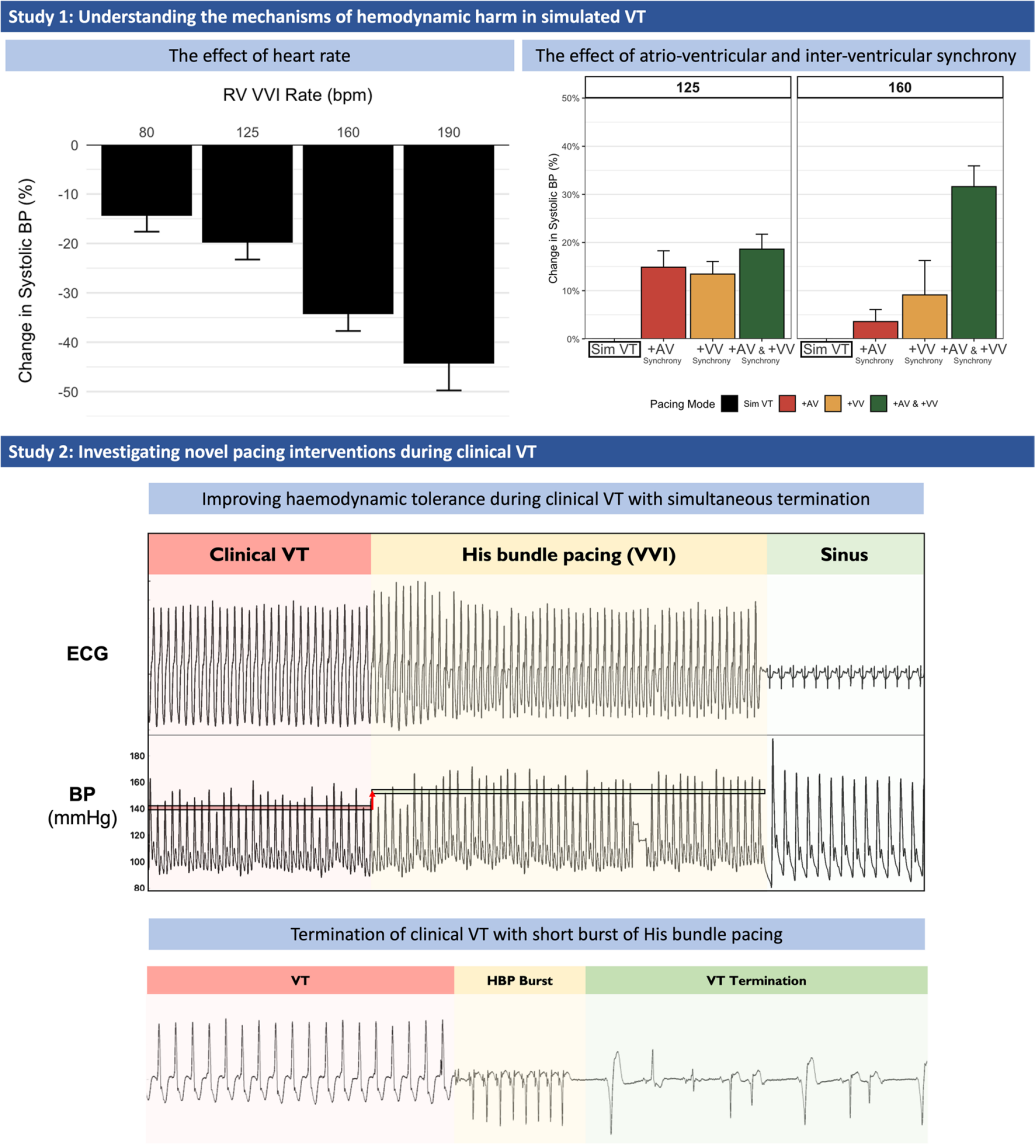

## Introduction

Hemodynamic compromise in ventricular tachycardia (VT) does not arise solely from rapid heart rates because sinus tachycardia and supraventricular tachycardias, at similar rates, generally cause less compromise. One contributor is the loss of atrial synchrony since it is reported that atrial contraction can contribute ~ 30% of ventricular filling [1–4]. Another contributor is the loss of ventricular synchrony [1, 5–7], since VT typically arises outside the normal conduction system responsible for coordinated ventricular activation.

If hemodynamic status during VT could be improved, this would allow VT to be better tolerated. This would potentially enable implantable cardioverter-defibrillator (ICD) shock therapies to be withheld for longer, allowing more time for VT to spontaneously terminate, thereby reducing the need for ICD shocks.

We investigate pacing interventions aimed at improving hemodynamic status during VT using a simulated VT paradigm and also using clinical VT.

Study 1 simulates VT with ventricular pacing, and separately examines the impact of restoring atrial and ventricular synchrony, testing the hemodynamic impact of these components.

Study 2 is hypothesis-generating and addresses the impact of restoring atrial and ventricular synchrony during true *clinical* VT in a small cohort.

## Methods

### Patient population

#### Study 1: understanding the mechanisms of hemodynamic harm during simulated VT

Patients with a left ventricular ejection fraction (LVEF) < 40% who either already had a cardiac resynchronization therapy device implanted (with leads positioned within the right atrium, His bundle, and right ventricle) or were attending for a clinically indicated invasive electrophysiology (EP) procedure (including VT stimulation/ablation procedures) were recruited with leads temporarily placed within the right atrium, His bundle and right ventricular (RV) apex.

#### Study 2: investigating novel pacing interventions during clinical VT

We investigated pacing interventions if VT was induced, with VT stimulation protocols, in the patients attending for an invasive EP procedure, including VT ablations.

We did not recruit patients who were in persistent atrial fibrillation or if patients were unable to give informed consent.

### Measurements

We simultaneously recorded a continuous 3-lead ECG (Fukuda Denshi 7100, Fukuda Denshi, Japan) and beat-by-beat blood pressure measured either invasively or, if not clinically indicated, non-invasively with the use of a Finometer (Finapres Nova, Finapres Medical Systems, NL). We report the measured average systolic blood pressure over 15 s in the reference state immediately prior to a pacing transition and the following 15 s after the transition (the reference state was either sinus rhythm or simulated VT in Study 1 and clinical VT in Study 2). For patients undergoing invasive EP procedures, quadripolar EP catheters were positioned in the high right atrium, His bundle, and RV apex.

These experiments were conducted during simulated VT in Study 1 to enable accurate quantification of the impact of the different pacing interventions. While artificial, this serves to enhance the mechanistic understanding for Study 2 in which we tested these therapies during clinical VT.

### Pacing protocols

#### Study 1: understanding the mechanisms of hemodynamic harm during simulated VT

The pacing protocol aimed to help delineate the relative hemodynamic effect of the following: (1) heart rate, (2) atrioventricular synchrony, (3) biventricular synchrony, and (4) combined atrioventricular and biventricular synchrony for patients with VT.

Thirty seconds of the patient’s intrinsic baseline rhythm were recorded before and after each episode of simulated VT.

**Fig. 1:**
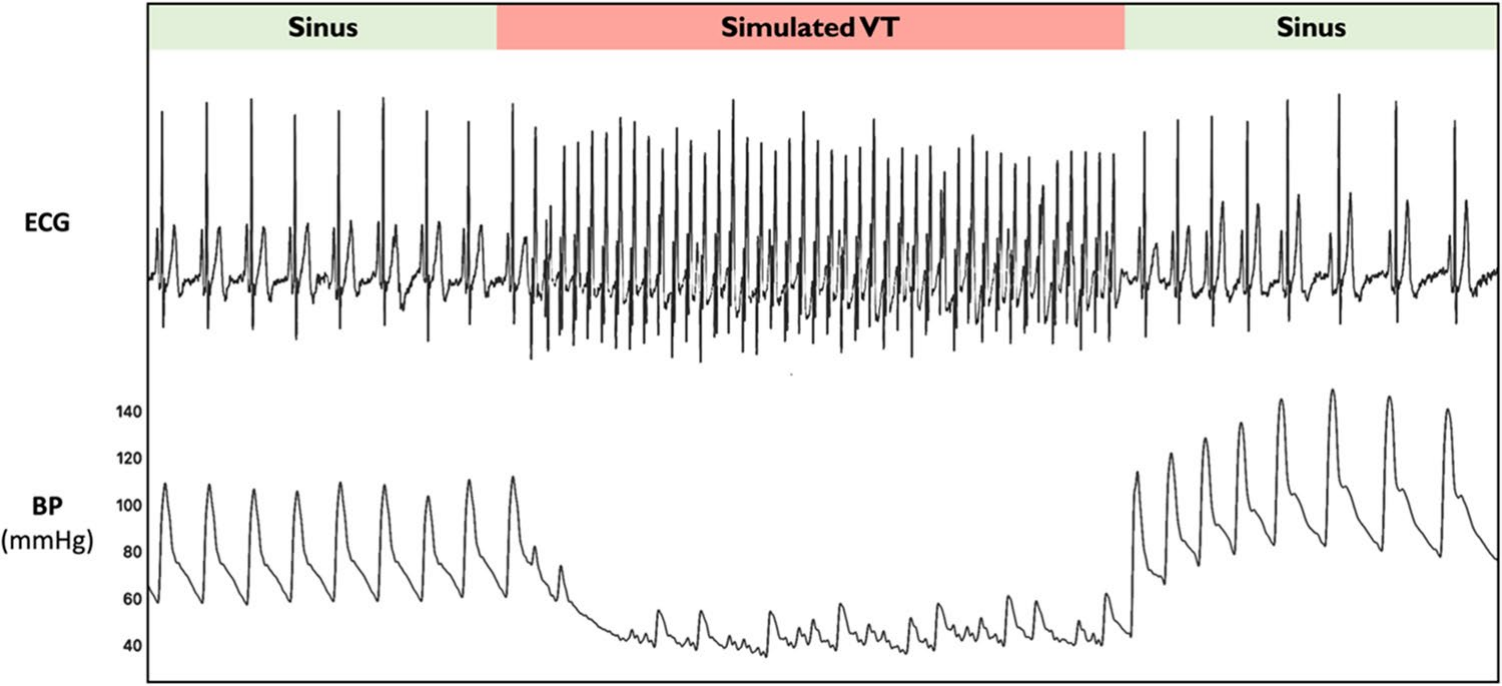
Example trace of the impact of simulated VT rate (160 bpm) on hemodynamics

Simulated VT was achieved by asynchronous ventricular pacing from the RV apex at various heart rates (80, 125, 160, and 190 bpm; Fig. 1). This resembled VT in that there was neither atrioventricular nor biventricular synchrony.

Example trace of the simultaneous ECG and beat-by-beat blood pressure recordings in a patient undergoing simulated VT generated from right ventricular apical pacing at a rate of 160 bpm with evidence of a profound change in hemodynamic status. BP, blood pressure; ECG, electrocardiogram; VT, ventricular tachycardia.

Participants underwent each of the pacing protocols three times, in a computer-generated random order.

To achieve restoration of atrioventricular synchrony alone, we performed synchronous dual chamber atrial and RV pacing at 125 bpm and 160 bpm (AV delay 140 ms).

Restoration of ventricular synchrony alone was performed by His bundle pacing at 125 bpm and 160 bpm in all patients. Standard His bundle capture criteria were utilized to confirm capture prior to the experimental protocol [8] and subsequently confirmed during the pacing maneuvers when the stimulus to ventricular electrogram interval was measured to confirm this reflected conduction down the His Purkinjee network. In all cases, non-selective capture was achieved.

Finally, restoration of atrioventricular and biventricular synchrony was achieved by synchronous dual chamber atrial and His bundle pacing at 125 bpm and 160 bpm (AV delay 100 ms).

For the restoration assessment protocols, asynchronous RV pacing at 125 or 160 bpm was performed for 20–30 s, and then the pacing mode was changed to determine the effect of the tested component on hemodynamic response.

#### Study 2: investigating novel pacing interventions during clinical VT

In Study 2, we aimed to assess the benefit derived through the correction of atrioventricular and biventricular synchrony during clinical VT. When VT did not occur spontaneously, induction approaches were utilized with a ventricular drive train and short coupled S2 and S3 beats as required.

Only patients who experienced spontaneous or induced VT after pace stimulation were included in this part of the study.

We allowed approximately 15 s of clinical VT before testing the pacing interventions described below. Where possible, three alternations were made between clinical VT and the tested pacing intervention.

The experimental pacing interventions we aimed to test included restoration of atrioventricular synchrony through the addition of atrial pacing at ~ 5 bpm above the clinical VT rate and attempted restoration of biventricular synchrony through the addition of His bundle pacing at ~ 5 bpm above the clinical VT rate. His bundle pacing confirmation approaches are detailed above.

It was not possible to try every pacing protocol in each patient. We prioritized His bundle pacing as this was both novel, and we felt that this had the greatest potential to deliver hemodynamic improvement. Following the unexpected termination of VT with His bundle pacing in some of the early study participants, the protocol was adapted to deliver a short burst of His bundle pacing (8–15 beats) at 91% of the tachycardia cycle length, aiming to be less aggressive than traditional anti-tachycardia pacing (ATP).

We measured the change in acute hemodynamic function relative to VT, for each of the performed interventions. During VT, we measured the stimulus timing in relation to the VT cycle length and the preceding ventricular beat to attempt to determine the ideal timing of stimuli to interact with the VT.

### Ethics

Ethical approval was obtained by the local Research Ethics Committee (London – Central 16/LO/0339). All participants provided written informed consent.

### Statistical analysis

#### Study 1: understanding the mechanisms of hemodynamic harm during simulated VT

To assess the impact of the rate of VT on the hemodynamic harm for each patient, we calculated the percentage drop in systolic blood pressure, for each rate, as compared to the patient’s baseline rate. Group differences were assessed using ANOVA.

The importance of atrioventricular and biventricular synchrony during VT were assessed as the potential benefit that might be achieved through their restoration as compared to simulated VT at the same heart rate. Calculations were made at 125 and 160 bpm. The percentage increase in systolic blood pressure with the restoration of either atrioventricular or biventricular synchrony alone or in combination was compared to simulated VT (without atrioventricular or biventricular synchrony) and quantified.

#### Study 2: investigating novel pacing interventions during clinical VT

To assess the impact of the pacing intervention on hemodynamics during VT, the percentage change in systolic blood pressure was calculated between the VT state and the pacing intervention. Using the mean change for each patient with each potentially therapeutic pacing approach, the mean across all patients was then calculated. The t-test was used to determine if the change was significantly different from zero.

## Results

### Study 1: understanding the mechanisms of hemodynamic harm during simulated VT

Twenty-four patients were recruited with a mean age of 68 ± 9 years and mean LVEF of 32 ± 7% (Table 1).

**Table 1:** Characteristics of the 24 patients included in Study 1

Characteristic	*N* = 24
Age (years)	68 ± 9
Male	19 (79%)
LV ejection fraction (%)	32 ± 7
Ischemic etiology of ventricular impairment	13 (54%)
Diabetes mellitus	6 (25%)
NYHA class	2.3 ± 0.5
BNP (ng/l)	568 ± 909
Resting systolic blood pressure (mmHg)	119 ± 15
Resting heart rate (bpm)	62 ± 7
QRS duration (ms)	113 ± 23

Mean ± SD; *N* (%)

*BNP* brain natriuretic peptide, *LV* left ventricular, *NYHA* New York Heart Association

#### Impact of VT rate on hemodynamics

**Fig. 2:**
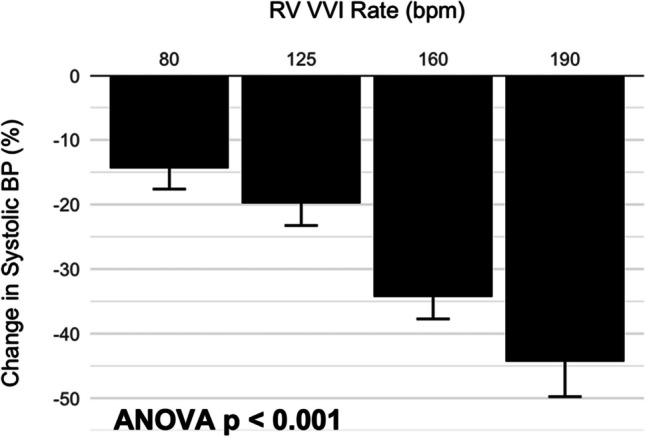
Impact of VT rate on hemodynamics. Percentage change in systolic blood pressure during simulated VT at different rates compared to baseline measurements. Error bars denote the confidence intervals. ANOVA, analysis of variance; BP, blood pressure; RV, right ventricular; VT, ventricular tachycardia

Each rate of simulated VT caused a significant fall in systolic blood pressure (*p* < 0.005) with the fall being larger at progressively higher heart rates (Fig. 2, ANOVA *p* < 0.0001 for difference between groups).

#### Restoration of atrial synchrony

**Fig. 3:**
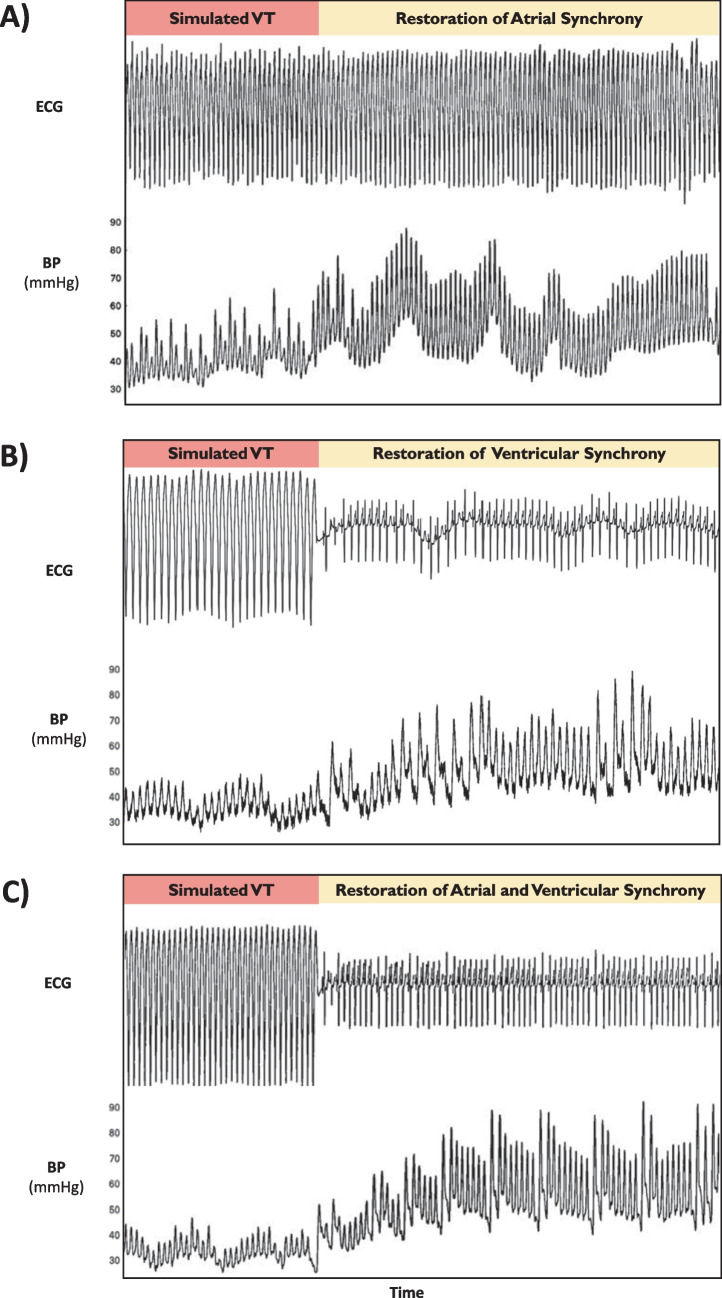
Hemodynamic effect during simulated VT when correcting for the loss of atrioventricular and ventricular synchrony (in isolation and combined). Examples of raw ECG and blood pressure traces demonstrating hemodynamic improvements when correcting for the loss of: **a** isolated AV dyssynchrony via RV DDD pacing (simulated VT rate 160 bpm), **b** isolated interventricular dyssynchrony via His VVI pacing (simulated VT rate 125 bpm). and **c** both combined via His DDD pacing (simulated VT rate 160 bpm). BP, blood pressure; ECG, electrocardiogram; RV, right ventricular; VT, ventricular tachycardia

**Fig. 4:**
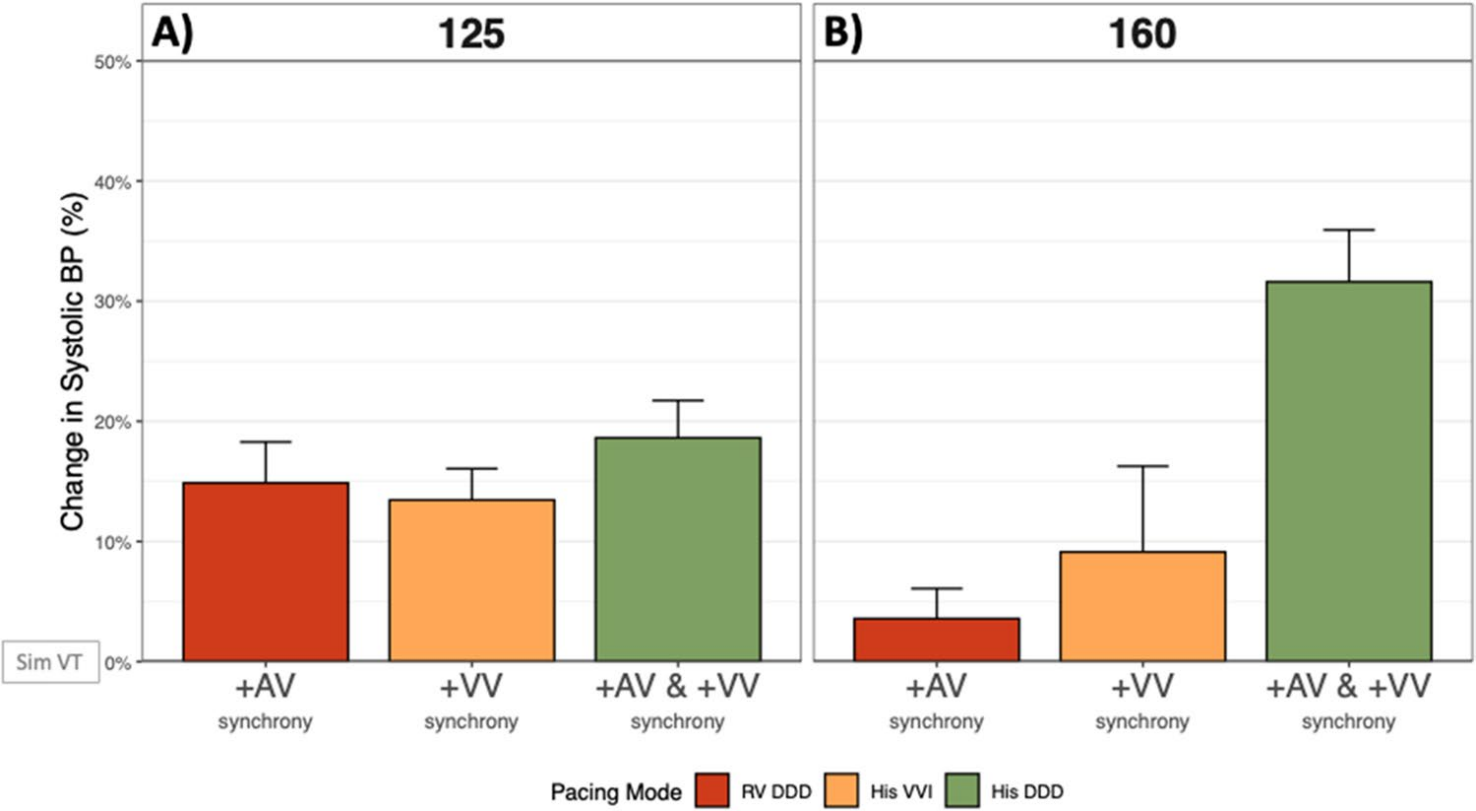
Mean change in acute hemodynamics during simulated VT when correcting for the loss of atrioventricular and ventricular synchrony. Mean change in acute hemodynamics in all tested patients when correcting atrioventricular synchrony (RV DDD), isolated biventricular synchrony (His VVI), and simultaneous atrioventricular and biventricular synchrony (His DDD) at rates of **a** 125 bpm and **b** 160 bpm. BP, blood pressure; Sim, simulated; VT, ventricular tachycardia

Compared with simulated VT, the addition of atrial synchrony (DDD: synchronous atrial and RV pacing) increased systolic blood pressure significantly by + 14.9% at 125 bpm (*p* = 0.0011) and by + 3.6% at 160 bpm (*p* = 0.2117, Fig. 3a and Fig. 4).

#### Restoration of ventricular synchrony

Compared with simulated VT, the addition of non-selective His bundle pacing to provide biventricular synchrony increased systolic blood pressure significantly by + 13.5% at 125 bpm (*p* = 0.0002) and by + 9.1% at 160 bpm (*p* = 0.242, Fig. 3b and Fig. 4).

#### Simultaneously correcting both contributors

Compared with simulated VT, the addition of simultaneous atrioventricular and biventricular synchrony using DDD (synchronous atrial and His bundle pacing) significantly improved systolic blood pressure by + 18.6% at 125 bpm (*p* < 0.0001) and by + 31.6% at 160 bpm (*p* = 0.0003, Fig. 3c and Fig. 4).

### Study 2: investigating novel pacing interventions during clinical VT

Eleven patients had spontaneous or induced VT. The characteristics of these 11 are shown in Table 2. The morphology in V1 of 10 patients was available and was negative in 5/10 with 3 patients having septal VT and positive in 5 patients in keeping with an left ventricular VT circuit.

**Table 2:** Baseline characteristics of 11 patients with spontaneous or induced VT (study 2)

Characteristic	*N* = 11
Age (years)	71 ± 10
Male VT rate (bpm)	10 (83%) 143 ± 21
LV ejection fraction (%)	27 ± 6
Ischemic etiology of ventricular impairment	8 (66%)
Diabetes mellitus	2 (17%)
NYHA class	2.4 ± 0.5
BNP (ng/l)	551 ± 287
QRS duration (ms)	111 ± 15

Mean ± SD; *N* (%)

*BNP* brain natriuretic peptide, *LV* left ventricular, *NYHA* New York Heart Association, *VT* ventricular tachycardia

Of the 11 VT episodes, 1 rapidly degenerated into ventricular fibrillation before the pacing protocol could be delivered. The His bundle pacing protocol was delivered in the remaining 10 patients. Due to clinically important time constraints, only 3 patients received the atrial pacing protocol.

#### Effect of His bundle pacing protocol in clinical VT

The first 6 patients received VVI His bundle pacing 5 bpm above the initial VT rate (~ 95% of the tachycardia cycle length) which increased systolic blood pressure by + 14.2%, (CI 7.8–20.6; *p* = 0.0023). Once stable, the interval from stimulus to QRS onset was 45 ± 18 ms, in keeping with ventricular activation via the His Purkinje system.

**Fig. 5:**
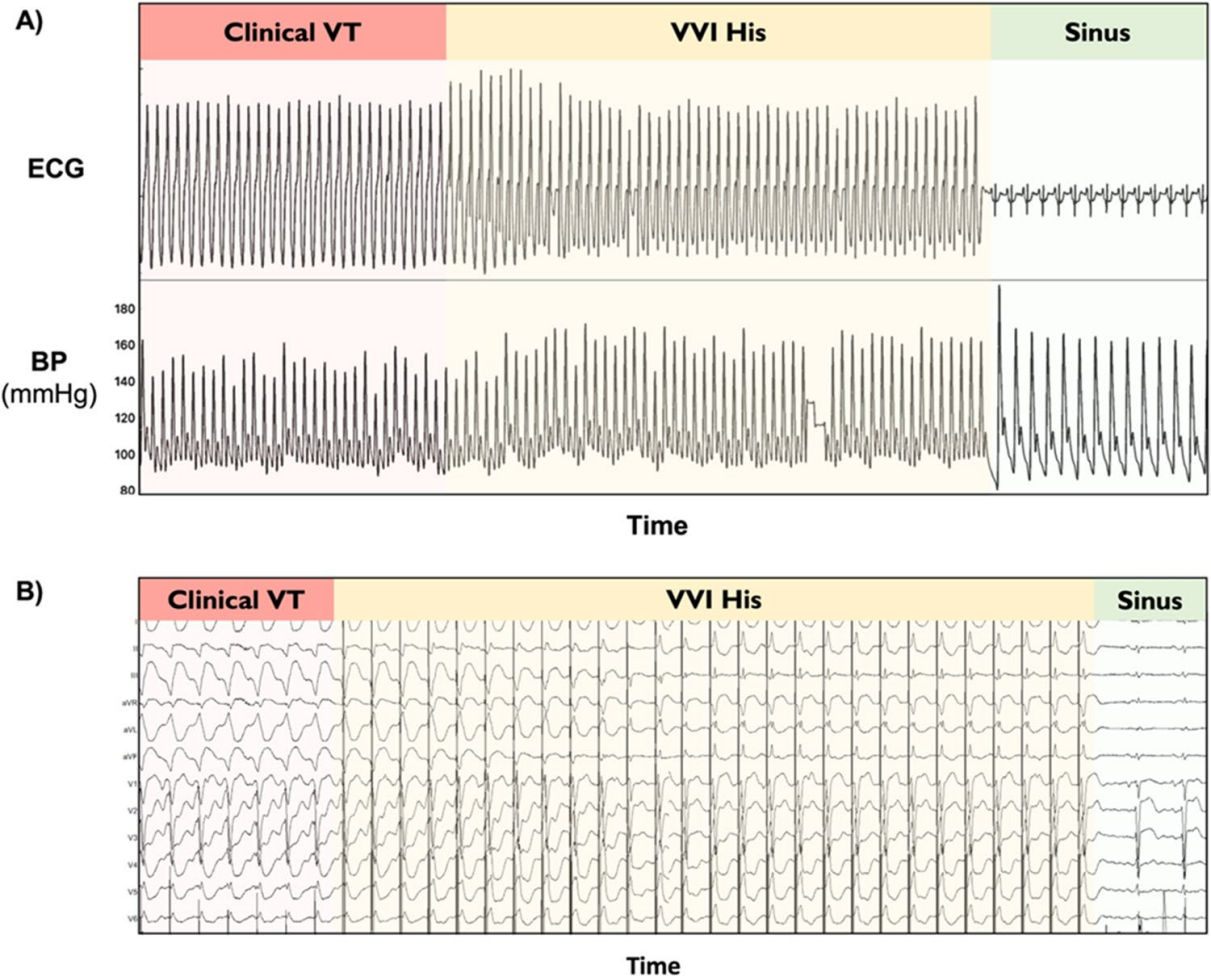
Hemodynamic improvement and termination of clinical VT by His VVI pacing. **a** The beneficial effect of His pacing on hemodynamics. **b** The progressive fusion and gradual assimilation of ventricular activation by His bundle pacing resulting in normalization of ventricular activation. This ultimately leads to termination of VT. BP, blood pressure; ECG, electrocardiogram; VT, ventricular tachycardia

In 2 of those first 6 patients, the VT was terminated by His pacing (Fig. 5). In a single patient, we performed dual chamber His bundle pacing during VT which resulted in a significant improvement in hemodynamics.

Because of the potential clinical utility of VT termination, we modified the protocol for the final 4 patients: We no longer aimed to measure the effect on systolic blood pressure, but rather the potential to terminate VT with a short burst of pacing at a shorter cycle length (~ 91% of TCL).

**Fig. 6:**
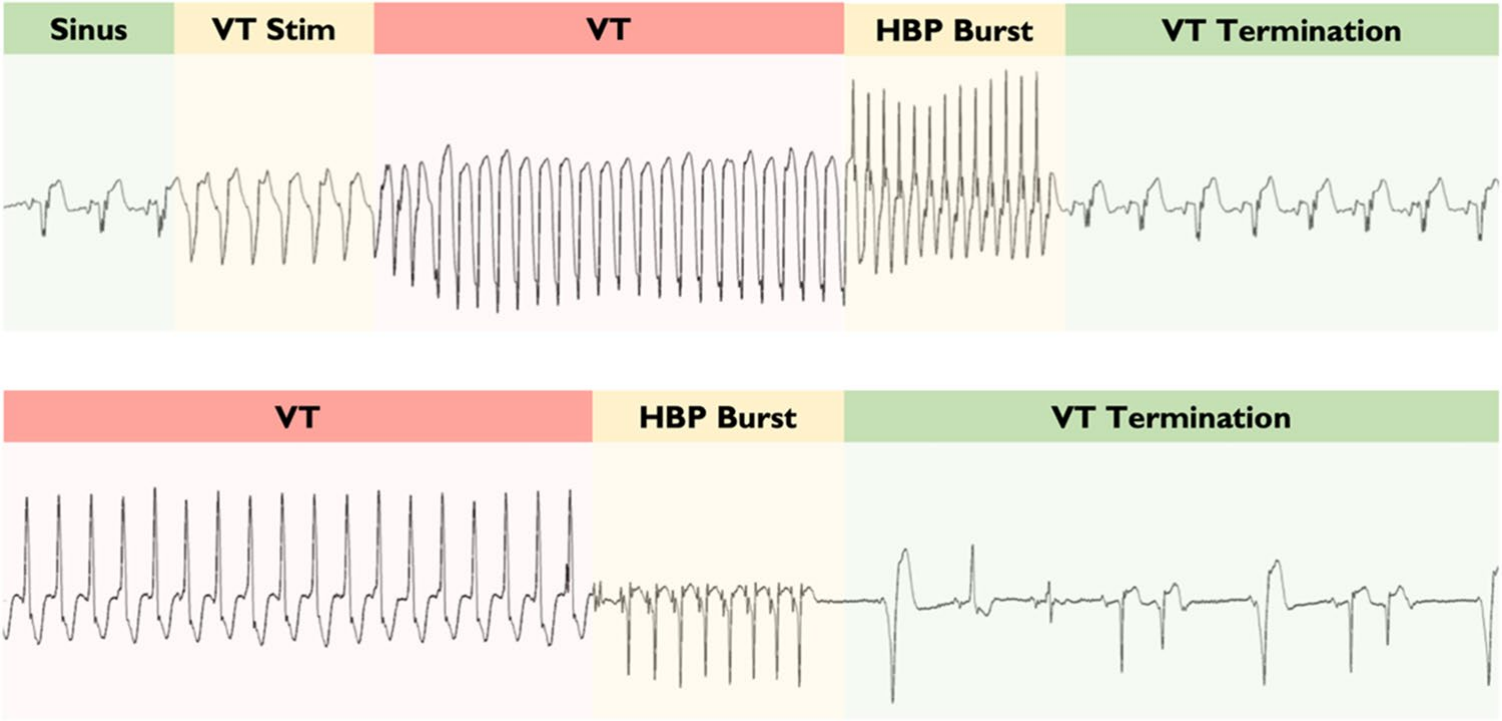
Clinical VT examples terminated with short bursts of His bundle pacing. Example traces of short bursts of His bundle pacing terminating episodes of clinical VT. HBP, His bundle pacing; VT, ventricular tachycardia

In the next 4 patients, we were able to terminate VT with a mean of 1.3 attempts, with all but one terminating on the first attempt (Fig. 6).

#### Effect of atrial pacing in VT

Three of the 10 patients underwent overdrive atrial pacing at 5 bpm above the VT rate. Acute hemodynamic function improved on average by 11.3% (*p* = 0.0017; CI 6.0–16.6).

**Fig. 7:**
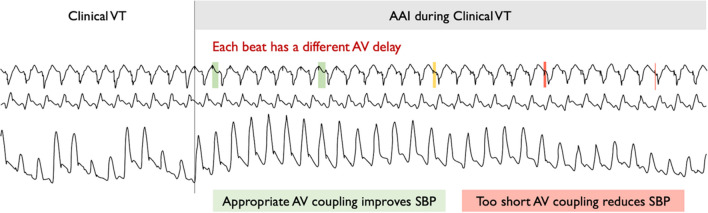
AAI pacing during clinical VT with varying AV delays. Sample trace of 18 s from the first of the 3 AAI pacing patients demonstrating the hemodynamic effect of varying AV delays during clinical VT. AV, atrioventricular; SBP, systolic blood pressure; VT, ventricular tachycardia

Because the VT rate was different to the atrial pacing rate, the timing of the atrial stimulus in relation to the QRS varied from beat to beat. We could therefore plot the increment in systolic blood pressure against the AV delay on a beat-by-beat basis. This revealed that there was a limited range of AV delays (AV delay > 60 ms) within which atrial pacing provided a net benefit; outside this range, it provided a net harm. In one patient, however, the pacing entrained the VT so that the range of AV delays was narrow (Fig. 7).

## Discussion

Through an improved understanding of the mechanisms of harm during VT, we have demonstrated that it might be possible to significantly improve hemodynamic function utilizing novel pacing interventions. These aim to reduce the harmful components of dyssynchrony which occur during VT.

These strategies could be used to improve hemodynamic stability and therefore tolerability of VT in a range of situations. This includes in the catheter laboratory during VT mapping and ablation, and in ICD recipients, who could potentially have therapies safely withheld for even longer than is currently prescribed.

### Evaluating mechanisms of harm

Although faster VTs generally cause more hemodynamic compromise, this is not because high rates themselves are so harmful. Fast rates can occur in sinus rhythm and atrial arrhythmias without such a profound hemodynamic deficit.

The reason that faster VTs cause more compromise is that VT is essentially always accompanied by both atrial and ventricular dyssynchrony. Atrial dyssynchrony is inevitable because the atrium is no longer driving the ventricular rate. The atrium might be activated at the same rate if there is retrograde conduction, but even then, the timing will generally be less than optimal. Ventricular dyssynchrony is universal except in the rare subset of fascicular VTs. Therefore, atrial and ventricular dyssynchrony will always have an adverse effect, but the impact may be smaller if the heart rate is relatively low. As the heart rate rises, the time available in the cardiac cycle for chamber filling and ejection becomes increasingly compressed so that the impact of dyssynchrony becomes proportionally more important [9].

### Potential applications in ICDs

Conventional ATP is typically delivered from the right ventricle. It is not always successful at terminating VT as often the pacing stimulation is coming from a site distant to the VT exit site. It can also worsen hemodynamics and occasionally even causes rhythm acceleration and hemodynamic collapse. Furthermore, while device therapy has an important role in the management of VT, landmark trials such as MADIT-RIT [10] and ADVANCE III [11] have shown that reducing therapies by only treating faster heart rates and delaying therapies leads to a reduction in mortality. This approach, however, potentially comes at the expense of some hemodynamically compromising arrhythmias having delayed treatment and slower ventricular tachycardias being left untreated.

The findings from our study suggest that we may be able to refine ICD therapies to address these deficiencies. The findings of our study could be applied with a new ICD therapy algorithm that aims to improve hemodynamic tolerability of VT through atrial and conduction system pacing at heart rates which are similar to the VT rate. This “VT stabilization algorithm” could potentially be applied to slower VTs, which are not presently treated with current primary prevention guideline programming recommendations. It would be unlikely to cause harm because atrial and conduction system pacing could be delivered at heart rates which are only slightly above the VT rate. The aim would be to maintain cardiac output during these slower VT episodes and allow time for these episodes to self-terminate. The new algorithm (which improves hemodynamic function) could also be applied to more rapid VT episodes, to allow longer detection windows to be programmed. By adjusting this setting, the requirement for shocks or ATP could be reduced by helping to sustain cardiac output during these longer detection windows. Ideally this algorithm would be used in conjunction with a hemodynamic sensor incorporated into the device to assess hemodynamic status [12]. This new algorithm requires further investigation prior to clinical application, but we think the findings of our study provide the justification to support this further work.

### Potential to terminate ventricular tachycardias

During clinical VT, restoring biventricular synchrony through His bundle pacing had two effects. Firstly, it significantly improved hemodynamics, despite pacing at a slightly higher heart rate than the clinical VT. Secondly, His bundle pacing showed an ability to terminate VT. This appears to be the first report of this. We propose two potential mechanisms for VT termination.

One mechanism is simply akin to that of conventional ATP delivered from the right ventricle. Even via this mechanism, the His bundle may be a better origin for activation because (a) the impulses come from a site closer to the VT circuit allowing it to readily enter the excitable gap (enabling peeling back of refractoriness) and (b) entering the conduction system enables more rapid propagation of the impulses.

An alternative mechanism is that when delivered, His bundle pacing rapidly activates the entire ventricle, while the VT wavefront is mired in a slow diastolic pathway and renders all the exit sites refractory, thus potentially enabling VT termination.

Whatever the mechanism, His bundle pacing provides better hemodynamic tolerability during this time.

### Focusing on restoring ventricular synchrony

We focused efforts on restoring ventricular synchrony rather than atrioventricular synchrony as it has the potential of being universally applicable. Some patients may not have an atrial lead or be able to capture atrial myocardium (e.g., due to the presence of atrial fibrillation), but all patients will be able to have ventricular stimulation. Conduction system pacing approaches have progressed from His bundle stimulation to left bundle branch area pacing. Future studies are needed to evaluate whether similar conduction system pacing effects are seen at the left bundle location. Furthermore, the location of the exit site of the VT may also be relevant for a pacing stimulus to optimally interact with the VT circuit. We have ongoing studies evaluating this.

Current defibrillator leads have already been considered for use in the left bundle area [13], and novel leads are being used, and testing proposed, for potential implantation in this area too [14]. These strategies may therefore make it possible in the future to improve hemodynamic tolerability and terminate VT, even with just a single chamber device.

If there is an atrial lead present, this may allow the additional potential benefit of treating atrioventricular dyssynchrony. We found that the timing of the delivery of the atrial stimulus was key. There is a window of atrioventricular relationship, unique to each patient, that produced an increment in hemodynamics. Outside this window, atrial pacing worsens hemodynamics.

The clinical application of utilizing atrial pacing would need to carefully time the atrial stimulation to ensure the delivery of stimuli at opportune times only.

### Limitations

This study relied on asynchronous RV pacing as a surrogate for VT in its mechanistic component (Study 1). True VT more frequently has a re-entry mechanism and originates within the left ventricle and therefore may behave differently to this surrogate, with additional confounders brought about by autonomic effects. We did not assess for the impact of vagal tone on hemodynamic response during our study. This has recently been shown to potentially have an impact in a retrospective analysis with a reported drop in sinus rate (vagal response) likely associated with a generalized vagal response and potentially enhanced vasodepressor impact affecting clinical hemodynamics [15]. There is however prior supporting evidence that RV pacing has similar hemodynamic effects to true clinical VT [16].

Much like the initial work of RV-based ATP [17], the rates of VT in this study were lower than those which are currently set to be treated by primary prevention devices. The principles from the earlier studies of RV-based ATP did extrapolate to faster rates, but whether this will be the case for conduction system pacing approaches during VT still needs to be investigated. The conduction system may become refractory at high pacing rates, with evidence of rate dependent block; however, this was not seen in this study.

In this study, subjects did not receive RV pacing or even biventricular pacing as a comparator for the effects of His bundle pacing during VT. His bundle pacing is less prone to causing ventricular dyssynchrony. For cases where His bundle pacing was delivered via an EP catheter, the extent of non-selectivity may be larger than that which might be expected from a deployed permanent pacing lead. This study did not evaluate the role of left bundle branch area stimulation which is now frequently being considered as a more pragmatic site for conduction system pacing [18]. While we might expect similar results from more distal conduction system capture, replication of this study should be performed from the left bundle location. This study predominantly enrolled patients with narrow QRS, while conduction system pacing can often reverse bundle branch block. It is unknown whether conduction system capture without reversal of bundle branch block would have similar hemodynamic benefit during VT.

This study only tested dual chamber pacing modes with fixed AV delays. It is conceivable though if AV delays were personalized utilizing an AV delay optimization approach that this could improve hemodynamics further than that already seen.

In this proof-of-concept study, not all subjects were put through every aspect of the protocol. When patients were found to terminate with His bundle pacing, we shortened the duration of His bundle pacing.

## Conclusions

Loss of atrial and ventricular synchrony in patients with LV impairment contributes importantly to hemodynamic harm in VT. During VT, His bundle pacing can significantly improve cardiac function. Furthermore, for the first time, we have documented that His bundle pacing can *terminate* VT.

Carefully timed pacing stimuli delivered at the His bundle appears to allow VT to be better tolerated or even terminated.
